# Knowledge gaps and future directions in cognitive functions in children and adolescents with primary arterial hypertension: A systematic review

**DOI:** 10.3389/fcvm.2022.973793

**Published:** 2022-10-20

**Authors:** Ignacio Lucas, Kristijonas Puteikis, Manish D. Sinha, Mieczysław Litwin, Kajus Merkevicius, Karolis Azukaitis, Rina Rus, Michał Pac, Lukasz Obrycki, Tonje Bårdsen, Joanna Śladowska-Kozłowska, Elif Sagsak, Empar Lurbe, Susana Jiménez-Murcia, Augustina Jankauskiene, Fernando Fernández-Aranda

**Affiliations:** ^1^Department of Psychiatry, University Hospital of Bellvitge, Barcelona, Spain; ^2^CIBER Fisiopatología de la Obesidad y Nutrición (CIBEROBN), Instituto de Salud Carlos III, Madrid, Spain; ^3^Psychoneurobiology of Eating and Addictive Behaviors Group, Neurosciences Programme, Bellvitge Biomedical Research Institute (IDIBELL), Barcelona, Spain; ^4^Faculty of Medicine, Vilnius University, Vilnius, Lithuania; ^5^Department of Paediatric Nephrology, Evelina London Children’s Hospital, Guy’s and St Thomas’ NHS Foundation Trust, London, United Kingdom; ^6^British Heart Foundation Centre, King’s College London, London, United Kingdom; ^7^Department of Nephrology, Kidney Transplantation and Hypertension, The Children’s Memorial Health Institute, Warsaw, Poland; ^8^Faculty of Medicine, Clinic of Pediatrics, Institute of Clinical Medicine, Vilnius University, Vilnius, Lithuania; ^9^Department of Pediatric Nephrology, Children’s Hospital, University Medical Centre Ljubljana, Faculty of Medicine, University of Ljubljana, Ljubljana, Slovenia; ^10^Department of Paediatric and Adolescent Medicine, Haukeland University Hospital, Bergen, Norway; ^11^Department of Pediatrics, University Children’s Hospital Heidelberg, Heidelberg, Germany; ^12^University of Health Sciences Turkey, Clinic of Pediatric Endocrinology, Gaziosmanpaşa Training and Research Hospital, Istanbul, Turkey; ^13^Department of Pediatric, Consorcio Hospital General, University of Valencia, Valencia, Spain; ^14^Department of Clinical Sciences, School of Medicine and Health Sciences, University of Barcelona, Barcelona, Spain

**Keywords:** blood pressure, primary arterial hypertension, executive functions, neuropsychology, pediatrics, childhood, adolescence, HyperChildNET

## Abstract

Arterial hypertension (AH) among adults is known to be associated with worse cognitive outcomes. Similarly, children and adolescents with AH could be expected to underperform during neuropsychological evaluations when compared with healthy peers. Our aims were to review the existing literature on cognitive functioning among children and adolescents with primary AH and to identify what additional evidence may be needed to substantiate the impact of hypertension on poor cognitive outcomes in this population. We conducted a systematic review of articles in PubMed and Web of Science published before 17 January 2022, reporting on cognitive testing among children and adolescents with primary AH. From 1,316 records, 13 were included in the review—7 used battery-testing while other employed indirect measures of cognitive functions. Most of the studies reported worse results among individuals with AH. Results of two prospective trials suggested that cognitive functioning may improve after starting antihypertensive treatment. Ambulatory blood pressure monitoring was shown to be more strongly related to cognitive testing results than office measures of blood pressure. Significant confounders, namely obesity and sleep apnea, were identified throughout the studies. Our review indicates that evidence relating AH with poor cognitive functioning among youth is usually based on indirect measures of executive functions (e.g., questionnaires) rather than objective neuropsychological tests. Future prospective trials set to test different cognitive domains in children and adolescents undergoing treatment for AH are endorsed and should consider using standardized neuropsychological batteries as well as adjust the assessing results for obesity and sleep disorders.

## Introduction

Executive functions are high order cognitive processes that are necessary when an automatic response to a situation is insufficient. This typically occurs when the brain needs to involve additional resources to adapt its response to complex demands (1). These cognitive processes are involved in planning or reasoning, and are important abilities that have been shown to be related to critical development of the brain during childhood and adolescence (2, 3). Executive functions can be evaluated with standardized neuropsychological tasks that assess different types of functions, such as cognitive flexibility, working memory, or response inhibition. These complex cognitive processes are strongly related to the prefrontal structures of the brain (4), and require a large amount of resources; thus, the activations associated with these processes imply high oxygen demands (5, 6). Therefore, good brain vascularization and the adequate perfusion are essential to achieve a good performance in these high order cognitive processes. Consequently, a healthy development of the prefrontal cortex (PFC) remains critical for the optimal development of an individual’s executive functions (7). As the PFC reaches its maturity later than the rest of the brain structures, both gray and the white matter of the PFC present significant changes during childhood and adolescence (2, 8, 9).

Arterial hypertension (AH) during childhood and adolescence could imply vascular disruptions in the brain, and more precisely, in the PFC. This would be associated with vascular cognitive impairment, presenting deficits in highly demanding mental processes (10). The brain is susceptible to damage by high blood pressure (BP), which may cause microinfarctions, white matter lesions, promote atrophy, and disrupt the neurovascular unit as well as the perivascular space (11, 12). It has been shown that AH is associated with worse cognitive outcomes among adults and new findings suggest that such a relationship also exists in children and adolescents (13, 14). It is important to highlight that AH without a known secondary cause (i.e., primary hypertension) is becoming more and more frequent among children and adolescents (15).

The topic is of great interest as early-onset AH could potentially determine cognitive health later in life (16). It could be expected that if the AH in children is not treated correctly, these subjects would be at higher risk of presenting cognitive decline at older ages, as well as neurological brain disorders, including Alzheimer’s disease (10, 17). AH may cause both acute-severe neurological complications as it is in case of hypertensive crisis, and chronic, subclinical impairment of neurocognitive function (18, 19). Thus, at a neurological level, their BP levels remain important not only for future cognitive development, but are also affecting their health and quality of life from an early age.

Consequently, early detection and treatment of primary AH in children and adolescents may be a relatively easy measure to decrease the risk of future cognitive impairment. However, it remains unclear which cognitive domains are most susceptible to being damaged in children with AH and which are merely consequences of cofounders associated to AH such as obesity or sleep disturbances (20, 21). Additionally, there is not much information about the possible positive effects of antihypertensive treatment on cognitive impairment in children with AH. Therefore, the main aim was to review the literature on cognitive impairment in children and adolescents with primary AH, highlighting current evidence and identifying potential gaps in the literature. Thus, our first specific objective was to summarize the results of anti-AH intervention on the executive functioning of children with AH. Secondly, to gather evidence about what type of AH and what BP measurements are more associated to cognitive impairments (e.g., primary/secondary hypertension, central/peripheral measures, ambulatory, etc.). And finally, to summarize the evidence on the possible association with cofounders.

## Methods

### Literature information sources and search terms

The review process was conducted according to the PRISMA guidelines (22). Two databases (PubMed and Web of Science) were used to obtain literature search of articles published up to 17 January 2022. The following search string was used, without any filters in PubMed: “[(hypertension[Title/Abstract]) OR (blood pressure[Title/Abstract])] AND [(child*[Title/Abstract]) OR (pediat*[Title/Abstract]) OR (young[Title/Abstract] OR (adolescent*[Title/Abstract])] AND [(cogn*[Title/Abstract]) OR (executive[Title/Abstract]) OR (memory[Title/Abstract])]” and Web of Science: “AB = [(hypertension OR “blood pressure”) AND (child* OR pediat* OR young OR adolescent*) AND (cogn* OR executive OR memory)].”

### Inclusion and exclusion criteria

The inclusion criteria were: (1) pediatric studies (only individuals aged <18 years), (2) studies containing a group with primary AH (studies that did not explicitly rule out secondary hypertension among their sample were also considered for inclusion) and (3) using any instrument to assess cognitive functions. The exclusion criteria were: (1) records in other languages than English, (2) studies including no identifiable group with hypertension (e.g., blood pressure measures used only as continuous variables), (3) undefined methodology of BP measurement, (4) studies of secondary AH and (5) the relationship between hypertension and cognitive outcomes is not reported.

### Study selection and data extraction

The articles were first screened by title, selected for inclusion and reviewed in full by I.L. and K.P. Data extracted were: sample, age, method of BP measurement, type of hypertension, method of cognitive assessment, type of analysis, major results. Discrepancies were dealt with by mutual agreement between the reviewers.

### Quality appraisal

Quality of reviewed literature was assessed by two independent reviewers using the Downs and Black checklist (23). Cross-sectional and longitudinal studies were assessed. This scale has been broadly used to assess the methodological quality of health studies. Any discrepancies between the two reviewers were mediated by a third reviewer (Supplementary Table 1). The evaluation found that the studies were largely based on valid and reliable methods with good outcome reports.

## Results

### Study selection

**FIGURE 1:**
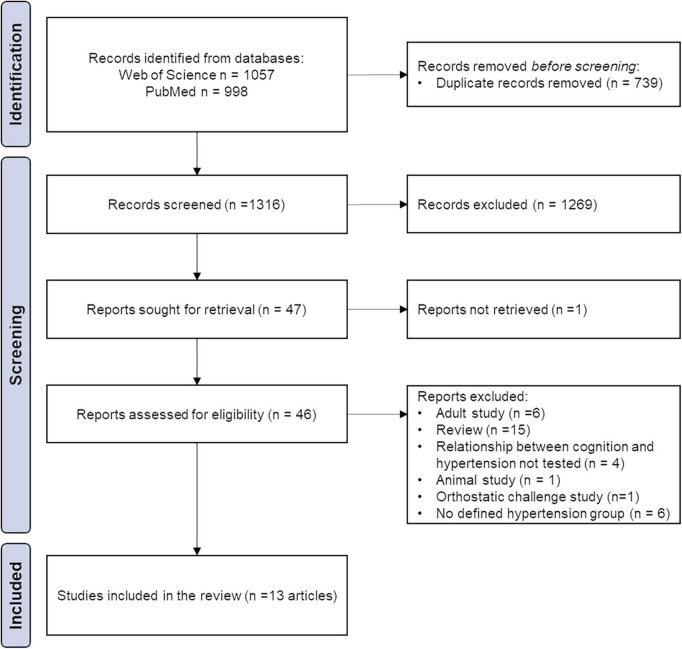
The article selection procedure, according to the PRISMA flowchart.

The search yielded 1,316 individual results from the two databases, of which 13 met the eligibility criteria (Figure 1). Additionally, six articles provided data on the relationship between BP and cognitive domains in the pediatric population but made no distinction of AH groups (24–29).

### Study results

A summary of available studies is presented in Table 1.

**TABLE 1:** Studies that evaluated cognitive impairment in children and adolescents with primary AH.

References	Study design	Sample size	n(AH)/n(HC)	Age (mean, SD or range)	Definition of hypertension	Type of arterial hypertension	Psychological assessment	Type of analysis	Main findings
Lande et al. (30)	Cross-sectional	5,077	288/4,789	6–16	Office BP, three measurements, ≥ 90th percentile	No distinction	WISC-R: block design (constructional skills) and digit span (short-term memory, attention, and concentration) subtests. WRAT-R: reading and arithmetic sections	Group comparisons and multiple linear regression	AH associated with lower Digit span after adjustment
Lande et al. (32)	Cross-sectional	64	32/32	16 (14–17)	History of office BP, three occasions, ≥ 95th percentile confirmed by 24 h-ABPM. WCH excluded	Only primary	IQ WISC (10–15) or WAIS (>15), CBCL, BRIEF	Group comparisons and multiple linear regression	CBCL: AH and BMI interaction on Internalizing behaviors BRIEF: Higher BRI, MI, and GEC scores in AH
Adams et al. (38)	Retrospective	201	100/101	15 (13–17)	Initial office BP ≥ 95th percentile, confirmation by 24 h-ABPM or three measurements	Only primary	Learning disabilities (defined as having a current individualized education plan or section 504 plan, both formal indicators of a student’s need for services to address an educational problem) or treated for ADHD	Categorical chi-square and multivariate analysis	AH associated with ADHD and LDs after adjustment
Lande et al. (33)	Prospective. 12-month follow-up of (32)	47	22/25	15 (13–16)	Repeated 24 h-ABPM at follow-up	Only primary	CBCL, BRIEF	Repeated measures ANCOVA	BRIEF: BRI, MI, GEC score improvement in AH
Ostrovskaya et al. (35)	Cross-sectional	14	n/a	14.5 (3.3)	24 h-ABPM	Only primary	BRIEF	Correlation analysis	Lower cerebrovascular reactivity slopes among AH, inversely associated with BRIEF scores
Lande et al. (34)	Cross-sectional	72	38/34	15.0 (2.1) [AH]	History of office BP, three occasions, ≥ 95th percentile confirmed by 24 h-ABPM	Only primary	SRBD-PSQ, CDI, BRIEF, RAVLT (attention, learning and memory), CPT-II (Attention and vigilance, Response inhibition, Attention), WASI: Vocabulary Matrix, Reasoning FSIQ (general intelligence), WISC-IV: Digit Span F and B, Spatial Span F and B (working memory, attention), DKEFS (Planning/Problem Solving), Tower test, Grooved Pegboard, CogState GMLT (Planning/Problem Solving, Memory), CogState Set Shifting	Group comparisons, correlation analysis	Higher SRBD-PSQ score associated with worse executive function (BRIEF), internalizing and externalizing behavior (CBCL), and worse ratings of depression (CDI)
Madaeva et al. (42)	Cross-sectional	38	n/a	14–17	Office BP, three occasions, ≥ 95th percentile	No distinction	Lichko’s Pathocharacterologic Diagnostic Questionnaire, “attention processes with Schulte tables, audio verbal features and visual-spatial memory by memorizing ten words and icons, peculiarities of speech and thinking with “60 words” and “classification of objects” techniques, making the story on the subject”	Group comparison within the hypertension group (with OSA or without)	Poorer cognitive outcome among adolescents with both AH and OSA
Lande et al. (31)	Cross-sectional	150	75/75	15.1 (2.2)	History of office BP, three occasions, ≥ 95th percentile confirmed by 24 h-ABPM	Only primary	BRIEF, RAVLT (attention, learning and memory), CPT-II (Attention and vigilance, Response inhibition, Attention), WASI: Vocabulary Matrix, Reasoning FSIQ (general intelligence), WISC-IV: Digit Span F and B, Spatial Span F and B (working memory, attention), DKEFS (Planning/Problem Solving), Tower test, Grooved Pegboard, CogState GMLT (Planning/Problem Solving, Memory), CogState Set Shifting	Group comparisons, correlation analysis, multivariate analysis	AH associated with worse verbal (RAVLT) and visual (CogState GMLT) learning and recall, verbal and visual reasoning (WASI). AH not impaired on tasks of vigilance and visuomotor reaction time, auditory and visual attention, working memory, problem solving, planning, set shifting
Kupferman et al. (40)	Cross-sectional, extension of (31)	150	75/75	15.1 (2.2)	History of office BP, three occasions, ≥ 95th percentile confirmed by 24 h-ABPM	Only primary	RAVLT (attention, learning and memory), WASI-Vocabulary Matrix (vocabulary), Grooved Pegboard (manual speed and dexterity), CogState GMLT (Planning/Problem Solving, Visual Memory)	Group comparisons, correlation analysis, multiple logistic regression	24 h-ABPM superior to office BP in distinguishing AH youth with lower neurocognitive test performance
Lande et al. (39)	Prospective. 12-month follow-up of (31)	121	55/66	n/a	History of office BP, three occasions, ≥ 95th percentile confirmed by 24 h-ABPM	Only primary	BRIEF, RAVLT (attention, learning and memory), CPT-II (Attention and vigilance, Response inhibition, Attention), WASI: Vocabulary Matrix, Reasoning FSIQ (general intelligence), WISC-IV: Digit Span F and B, Spatial Span F and B (working memory, attention), DKEFS (Planning/Problem Solving), Tower test, Grooved Pegboard, CogState GMLT (Planning/Problem Solving, Memory), CogState Set Shifting	Repeated measures ANCOVA	A year of anti-AH therapy did not improve neurocognitive test performance beyond improvement in control subjects. Anti-AH therapy may influence test performance by enabling strategic learning
Chrysaidou et al. (37)	Cross-sectional	116	38/78	11.20 (3.08)	Office BP, three measurements, ≥ 95th percentile + 24 h-ABPM	Only primary	BRIEF	Group comparisons, correlation analysis, multiple linear regression	Executive function lower in night-time AH, mediated by SBP despite obesity. Office-BP parameters inferior to 24 h-ABPM
Stabouli et al. (41)	Cross-sectional	92	46 (primary AH)/46 (secondary AH)	11.4 (2.9) [AH]	Office BP, three measurements, ≥ 95th percentile + 24 h-ABPM + cBP scores ≥ 95th	Compared primary vs. secondary hypertensive groups	BRIEF	Group comparisons, multiple linear regression	cSBP the only significant predictor of the parent MI. cSBP and BMI associated with parent BRI, but with an interaction between AH group and anti-AH treatment. Ambulatory AH group not predictive of BRIEF
Stabouli et al. (36)	Cross-sectional	99	30/69	11.1 (3.1)	Office BP, three measurements, ≥ 95th percentile + 24 h-ABPM	Only primary	BRIEF	Group comparisons, correlation analysis, ANCOVA, mediation analysis	Serum uric acid concentration associated with worse executive function, association partly mediated by night time SBP

ABPM, Ambulatory blood pressure monitoring; ADHD, attention deficit hyperactivity disorder; AH, arterial hypertension; ANCOVA, Analysis of covariance; BMI, body mass index; BRI, Behavior Regulation Index; BRIEF, Behavior Rating Inventory of Executive Function; cBP, central blood pressure; CBCL, Child Behavior Checklist; cSBP, central systolic blood pressure CogState; CDI, Child Depression Inventory; FSIQ, Full Scale Intelligence Quotient; GEC, Global Executive Composite; GMLT, CogState Groton Maze Learning Test; CPT-II, Conners’ Continuous Performance Test II; DKEFS, Delis-Kaplan Executive Function System; HC, healthy control; LD, learning disability; MI, Metacognition Index; OSA, obstructive sleep apnea; RAVLT, Rey Auditory Verbal Learning Task; SBP, systolic blood pressure; SRBD-PSQ, Pediatric Sleep Questionnaire: Sleep-Related Breathing Disorder scale; WASI, Wechsler Abbreviated Scales of Intelligence; WCH, white coat hypertension; WISC, Wechsler Intelligence Scale; WRAT, Wide Range Achievement Test.

#### Executive functions assessment

Neurocognitive assessment was heterogeneous, with seven studies reporting results of battery-testing, while indirect measures of executive functioning were evaluated in ten studies. Some of the validated tasks for direct assessment of the executive functions reported deficits in executive functioning in children with AH. Children with AH showed lower digit span in the verbal scale of the WISC-R, a measure of short-term memory, attention and concentration (30). Children with AH also reported worse performance than normotensive ones in the Rey Auditory Verbal Learning Test (RAVLT) and in the CogState Groton Maze Learning Test delayed recall, measures of short term and working memory (31). Additionally, AH was also related to worse performance in the Grooved Pegboard dominant hand subtest, showing less fine motor dexterity, and lower scores in the vocabulary subscale of the Wechsler Abbreviated Scales of Intelligence (31).

Six studies included in this review only assessed cognitive performance by indirect measures of executive functions and behaviors. The BRIEF questionnaire (Behavior Rating Inventory of Executive Function), a subjective proxy-reported measure for evaluating behaviors related to the executive functioning of these children, reported association between AH and higher scores in the Metacognition Index (MI), Behavioral Regulation Index (BRI) and Global Executive Composite (GEC) scales (32). High scores in the BRIEF scales suggest that there could be difficulties in their executive functions (32–37). Moreover, child behaviors have also been assessed using the Child Behavior Checklist (CBCL), a tool aimed at evaluating their internalizing and externalizing behaviors. AH in children was associated with both clinically significant higher internalizing and externalizing behaviors (32, 34). Also, in one of the studies, parents were asked about the presence of learning difficulties and children with AH were more likely to present learning difficulties than normotensive children (38).

#### Effect of anti-arterial hypertension treatment

Except for two trials of prospective design and one collecting retrospective data (38), the reports were cross-sectional. Prospective designs evaluated the effect of anti-AH therapy on the neurocognitive performance of children with AH. The results of one of the prospective studies reported that children with AH showed improvement in the BRIEF scale after 12 months of anti-hypertensive treatment (33). However, in the other prospective study, a year of anti-AH therapy did not improve neurocognitive test performance, but complementary analysis showed that it may have enabled children with AH to benefit from task learning at the same level as normotensive children (39).

#### Arterial hypertension etiology and measurements

Two studies used solely office BP measures to define AH and control groups, others sought confirmation by performing 24-h ambulatory blood pressure monitoring (ABPM). ABPM was found to be more precise in distinguishing hypertensive children with executive dysfunctions (37, 40). Ten of the studies excluded the participants with secondary hypertension from the study, only two studies did not make the distinction between primary and secondary hypertension and one study compared a group of children with primary AH to children with renal AH. This study observed that children with renal AH presented lower executive function performance but also higher levels of central SBP, and, after adjustment by their central SBP levels, no significant differences were found in parents’ cognitive and behavioral assessments (41). Besides, effects of systolic vs. diastolic AH was assessed in one study, which reported closer association of cognitive deficits with systolic AH (30).

#### Potential cofounders

The reviewed literature also analyzed the possible effect of cofounders associated to AH. One study found that the executive impairments were independent of obesity levels in children with AH (37). While others found that body mass index (BMI) was associated to the BRI scale of the BRIEF inventory (41) and internalizing behaviors of the CBCL (32). Also, one study found a positive association between higher levels of serum uric acid and worse executive performance (36). Furthermore, children with AH were more prone to sleep disordered breathing (34) and children with AH who also presented obstructive sleep apnea showed poorer cognitive performances (42). Interaction between disordered sleep and poorer executive functioning was also observed in children with AH (31). Additionally, one study showed that learning difficulties were independent from the presence of attention deficit hyperactivity disorder (ADHD) symptomatology (38).

#### Assessment instruments

After reviewing the literature on cognitive functions in children and adolescents with AH, we found that the used instruments were heterogeneous. Therefore, in Supplementary Table 2 we suggest a battery of instruments for evaluating cognitive functions in children and adolescents with AH. This battery includes a series of validated instruments (some of them used in the reviewed studies) that assess different cognitive domains and would allow easier comparison of the results.

## Discussion and future directions

The current review outlines the scarcity of studies that assess a wide spectrum of cognitive domains in children and adolescents with AH. There is a broader literature on the association of AH mediated cognitive impairments in adults, but fewer studies on children and adolescents (13, 16). Alongside reports relating global IQ and BP measurements, the few studies that employed neuropsychological batteries provide evidence that AH-associated cognitive damage may be generalized and reflected by deficits in verbal and visual reasoning, learning and recall, working memory and semantic/letter fluency. As impaired executive functions are known among adults with AH, this association could also be expected among children and adolescents as well (16, 43). However, a direct assessment of executive function (e.g., the CogState GMLT) has only rarely been used in children with AH (31). Identification of domain-specific damage could lead to better understanding on how neurovascular beds differ in their susceptibility to AH among pediatric and adult patients, and define the possible reversibility of these effects. For instance, it has been shown by Lande et al. that poor BP control limits the ability to use previous learning techniques for better performance during subsequent cognitive testing (39). The authors associate this finding with cognitive deficits that may remain present in adulthood. Thus, it is essential to further investigate: (1) which cognitive domains are most susceptible to damage by high BP in early life and (2) whether good BP control suffices for patients with AH to match controls in cognitive outcomes (otherwise, what other treatment, rehabilitation or learning techniques could improve their cognitive functions).

Globally, the reviewed studies report that children and adolescents with AH perform worse than their normotensive peers following a wide-range of neurocognitive domains. However, the investigations were very heterogeneous in their approach toward neurocognitive testing and therefore hamper the general extrapolating from the studies. Overall, the neurocognitive assessment using validated batteries reported deficits in executive functions in children and adolescents with AH compared to normotensive controls. According to the reviewed literature, young individuals with AH might have deficits in short-term memory, working memory, attention, concentration, fine motor dexterity and vocabulary (30, 31). However, the literature using valid direct assessment of executive functions in children and young people with AH is limited. Furthermore, several studies relied solely on subjective measures such as BRIEF and/or CBCL questionnaires to measure cognitive functions (namely executive domains, externalizing and internalizing behaviors). While BRIEF has good psychometric properties and ecological validity, it remains an indirect measure of executive functions that relies on patient or parent reporting of the child’s behavior in different environments (44). This may be especially problematic if subtle subclinical deficits are sought to be assessed, for which specialized tests of executive function are needed. Also, results obtained from BRIEF differ from recent studies showing that AH-related executive dysfunctions are either absent following adjustment for confounders (especially disordered sleep) or related only to measures of central but not brachial BP (31, 41). Finally, as BRIEF relies on parent reporting, it is also prone to additional bias if treatment groups are not blinded (as parents expect treatment to have effect on their child’s performance) (33).

As has been observed in this review, the literature that analyzed the possible effects of anti-AH treatment on the executive functions of children with AH is scarce. The few evidence measuring the effect of an anti-AH treatment seems to indicate that it could improve cognitive outcomes, but further research is needed. One of the few studies that analyzed the effect of the treatment found improvements in the executive functions of children with AH after 12 months of anti-AH therapy (33). However, the same research group was not able to replicate the findings in another study (39), although further analysis of the data of this second study showed that treatment could have led to improvements in children with AH similar to those of normotensive children. Moreover, most of the studies included in this review present cross-sectional designs that assess AH and executive functioning at a specific point of time. Therefore, it would be necessary to perform further longitudinal studies to evaluate if the cognitive deficits observed in children with AH in comparison to normotensive controls could be reduced following specific treatment or and/or reduction of BP levels.

The observation that the influence of higher BP on executive functions may be better detected through testing of central hemodynamics is in line with current pathophysiological theories suggesting that the latter are more relevant for AH mediated organ damage (45). Furthermore, the reviewed literature emphasizes that ABPM is the best method for detecting AH associated with possible cognitive deficits, and should therefore be the preferred measure to use in this clinical domain dysfunctions (37, 40). In this line, the measure of systolic AH was also more related to cognitive deficits than diastolic hypertension (30). Although neurological complications, both acute, clinically symptomatic and chronic, and subclinical are well described in children with secondary AH, from a public health point of view the important problem is the recognition of neurocognitive impairment in children and adolescents with primary AH. The cognitive differences between primary and secondary AH could be influenced by the general higher levels of BP of patients with secondary AH (41). Therefore, the analyzed publications emphasize the importance of a precise definition of the etiology of the AH and the employed measurement method.

Furthermore, pediatric studies investigating cognitive functions in AH face great methodological challenges in addressing confounders that may refute the hypothesis of a direct association between AH and worse performance during neuropsychological testing. While multiple regression and mediation analyses can help to partly distinguish the role of high BP in cognitive dysfunction, factors like comorbid sleep and mood disorders, ADHD symptomatology, as well as widespread obesity remain major reasons to question the direct relationship between cardiovascular and cognitive variables (34). Besides, studies treating learning difficulties and ADHD as end-points may miss mild early-onset cognitive decline that does not produce significant learning difficulties (38). The executive deficits can be associated with attentional problems in children with AH, however, children with ADHD seem to present lower levels of BP (46), so the deficits related to AH could be independent from the ADHD symptomatology. Recently, it has also been shown that uric acid levels alongside high BP are associated with worse executive functions (36). Such findings highlight the need for a complex approach toward studies of cognitive function in children with AH that would include biological as well as imaging biomarkers as part of the investigation process (13).

## Conclusion

Studies assessing cognitive function in children and adolescents with AH remain scarce and are highly heterogeneous in methodology. Most of the studies that assessed executive functions in this population widely relied on indirect parent-reported measures, with the limitations and possible biases that this entails. While several studies report deficits of executive functions among patients with AH, the improvement of cognition after treatment remains undetermined. Given the mixed findings, future studies should prospectively employ comprehensive neuropsychological batteries that directly assess different cognitive domains, including verbal/visual memory, working memory, attention, reaction speed and global intellect. The heterogeneity of the evaluation methods found in this review highlights the necessity of establishing a standardized protocol for evaluating the cognitive functioning of children with primary AH.

## Data availability statement

The original contributions presented in this study are included in the article/Supplementary material, further inquiries can be directed to the corresponding author/s.

## Author contributions

IL and KP conducted the review of the studies and wrote the first draft of the manuscript. IL, KP, EL, SJ-M, AJ, and FF-A contributed to the idea and conceptualization of the review. AJ and FF-A supervised the preparation of the review and the interpretation of the results. MS, ML, KM, KA, RR, MP, LO, TB, JŚ-K, ES, EL, and SJ-M revised the manuscript and provided substantial comments. All authors contributed to the article and approved the submitted version.
