# Supplementary material for: Knowledge gaps and future directions in cognitive functions in children and adolescents with primary arterial hypertension: A systematic review
Source: Front Cardiovasc Med. 2022 Oct 20;9:973793. doi: 10.3389/fcvm.2022.973793 (PMC9631488; doi:10.3389/fcvm.2022.973793)
Supplement: Supplementary file 1 [file Data_Sheet_1.docx]

**Supplementary Table 1**. Quality appraisal of the reviewed studies using the Downs and Black (1998) checklist.

|  | **1** | **2** | **3** | **4** | **5** | **6** | **7** | **8** | **9** | **10** | **11** | **12** | **13** | **14** | **15** | **16** | **17** | **18** | **19** | **20** | **21** | **22** | **23** | **24** | **25** | **26** | **27** |
| --- | --- | --- | --- | --- | --- | --- | --- | --- | --- | --- | --- | --- | --- | --- | --- | --- | --- | --- | --- | --- | --- | --- | --- | --- | --- | --- | --- |
| Lande et al., 2003 | 1 | 1 | 1 | N/A | 2 | 1 | 1 | N/A | N/A | 1 | 0 | 0 | N/A | N/A | N/A | 1 | N/A | 1 | N/A | 1 | N/A | N/A | N/A | N/A | 0 | N/A | N/A |
| Lande et al., 2009 | 1 | 1 | 1 | N/A | 2 | 1 | 1 | N/A | N/A | 1 | 0 | 0 | N/A | N/A | N/A | 1 | N/A | 1 | N/A | 1 | N/A | N/A | N/A | N/A | 1 | N/A | N/A |
| Adams et al., 2010 | 1 | 1 | 1 | N/A | 2 | 1 | 1 | N/A | N/A | 1 | 0 | 0 | N/A | N/A | N/A | 1 | N/A | 1 | N/A | 1 | N/A | N/A | N/A | N/A | 1 | N/A | N/A |
| Lande et al., 2010 | 1 | 1 | 1 | 1 | 2 | 1 | 1 | 0 | 1 | 1 | 0 | 0 | 1 | 0 | 0 | 1 | 1 | 1 | 1 | 1 | 0 | 0 | 0 | 0 | 1 | 0 | 5 |
| Lande et al., 2015 | 1 | 1 | 1 | N/A | 2 | 1 | 1 | N/A | N/A | 0 | 0 | 0 | N/A | N/A | N/A | 1 | N/A | 1 | N/A | 1 | N/A | N/A | N/A | N/A | 0 | N/A | N/A |
| Ostrovskaya et al., 2015 | 0 | 1 | 1 | N/A | 1 | 1 | 1 | N/A | N/A | 1 | 0 | 0 | N/A | N/A | N/A | 1 | N/A | 1 | N/A | 1 | N/A | N/A | N/A | N/A | 0 | N/A | N/A |
| Madaeva et al., 2016 | 1 | 1 | 1 | N/A | 2 | 1 | 1 | N/A | N/A | 0 | 0 | 0 | N/A | N/A | N/A | 1 | N/A | 1 | N/A | 1 | N/A | N/A | N/A | N/A | 0 | N/A | N/A |
| Lande et al., 2017 | 1 | 1 | 1 | N/A | 2 | 1 | 1 | N/A | N/A | 1 | 0 | 0 | N/A | N/A | N/A | 1 | N/A | 1 | N/A | 1 | N/A | N/A | N/A | N/A | 1 | N/A | N/A |
| Kupferman et al., 2018 | 1 | 1 | 0 | N/A | 1 | 1 | 1 | N/A | N/A | 0 | 0 | 0 | N/A | N/A | N/A | 1 | N/A | 1 | N/A | 1 | N/A | N/A | N/A | N/A | 1 | N/A | N/A |
| Lande et al., 2018 | 1 | 1 | 1 | 1 | 2 | 1 | 1 | 0 | 1 | 1 | 0 | 0 | 1 | 0 | 0 | 1 | 1 | 1 | 1 | 1 | 0 | 0 | 0 | 0 | 1 | 0 | 5 |
| Chrysaidou et al., 2020 | 1 | 1 | 1 | N/A | 2 | 1 | 1 | N/A | N/A | 0 | 1 | 0 | N/A | N/A | N/A | 1 | N/A | 1 | N/A | 1 | N/A | N/A | N/A | N/A | 1 | N/A | N/A |
| Stabouli et al., 2020 | 1 | 1 | 1 | N/A | 2 | 1 | 1 | N/A | N/A | 1 | 0 | 0 | N/A | N/A | N/A | 1 | N/A | 1 | N/A | 1 | N/A | N/A | N/A | N/A | 1 | N/A | N/A |
| Stabouli et al., 2021 | 1 | 1 | 1 | N/A | 2 | 1 | 1 | N/A | N/A | 1 | 1 | 0 | N/A | N/A | N/A | 1 | N/A | 1 | N/A | 1 | N/A | N/A | N/A | N/A | 1 | N/A | N/A |

**Supplementary Table 2.** Suggested battery of instruments for evaluating cognitive functions in children and adolescents with AH.

| **Cognitive function** | **Instrument** | **Reference** |
| --- | --- | --- |
| General Intelligence | Wechsler Intelligence Scale for Children – 5th Ed. (WISC-V) | Wechsler et al., 2015 (47) |
| Short term memory / Working memory | WISC-V: Digit/Spatial span tasks (Forward and Backwards) | Wechsler et al., 2015 (47) |
| Non-verbal intelligence | Raven Progressive Matrices | Raven & Raven, 2003 (48) |
| Visual learning | Cogstate - Groton Maze Learning Test (GMLT) | Snyder et al., 2005 (49) |
| Verbal learning | Rey Auditory Verbal Learning Test (RAVLT) | Schmidt, 1996 (50) |
| Cognitive Flexibility | Wisconsin Card Sorting Test (WCST) | Heaton, 2003 (51) |
| Response inhibition / Attention | Conners Continuous Performance Test 3rd Ed. (CPT-3) | Conners, 2014 (52) |
| Fine motor dexterity | Grooved Pegboard Test | Lafayette Instrument, 2015 (53) |
| Subjective assessment of cognitive, emotional, and behavioral functions | Behavior Rating Inventory of Executive Function - Parent form (BRIEF) | Gioia et al., 2000 (54,55) |
| Subjective assessment of cognitive, emotional, and behavioral functions | Behavior Rating Inventory of Executive Function - Self Report Version (BRIEF-SF) | Guy et al., 2004 (56) |
| Subjective assessment of child behaviour | Child Behavior Checklist (CBCL) | Achenbach & Rescorla, 2001 (57) |
